# Impact of labetalol versus nifedipine treatment on readmission risk in postpartum hypertension: A randomized controlled trial

**DOI:** 10.1002/pmf2.70005

**Published:** 2025-04-07

**Authors:** Todd Lovgren, Ruofan Yao, Brendan Connealy, Robert Bonebrake, Hemant Satpathy, Emily Patel, Joshua D. Dahlke

**Affiliations:** ^1^ Nebraska Methodist Women's Hospital and Perinatal Center Omaha Nebraska USA; ^2^ Division of Maternal‐Fetal Medicine Loma Linda School of Medicine Loma Linda California USA

**Keywords:** antihypertensive, hypertension, labetalol, nifedipine, postpartum, preeclampsia, pregnancy, readmission

## Abstract

**Importance:**

Postpartum hypertensive disorders are a leading cause of maternal mortality. Observational trials suggest outcomes between labetalol and nifedipine may not be similar, and further study is needed to determine optimal postpartum treatment.

**Objective:**

Compare readmission rates for hypertensive complications between two commonly used antihypertensives in pregnancy, labetalol and nifedipine.

**Design:**

Open‐label randomized controlled trial comparing labetalol and nifedipine for treatment of postpartum hypertension. The study was conducted from 2022 to 2024. Inclusion criteria: patients with two hypertensive blood pressures (> 140 systolic or > 90 diastolic) 4 h apart during their admission for delivery.

**Setting:**

Single‐center study in a Level III Obstetric hospital.

**Participants:**

A total of 3015 patients met the inclusion criteria during the study period and 323 patients over the age of 19 were enrolled and randomized to nifedipine or labetalol. Patients were excluded if already taking two antihypertensive medications and were discharged the day they met the criteria or had contraindications to study medications.

**Intervention:**

Patients were randomly assigned to nifedipine XR 30 mg twice daily or labetalol 200 mg every 8 h with the remainder of postpartum management at the discretion of their primary provider. Records were reviewed to identify readmissions due to hypertensive complications within 6 weeks of delivery.

**Main outcomes and measures:**

Our primary outcome was readmission due to hypertensive complications within 6 weeks of delivery utilizing intention‐to‐treat analysis.

**Results:**

Among 323 patients randomized, the labetalol group (*n* = 161) had higher BMI (35.3 vs. 33.5), greater GA at delivery (38 vs. 37 weeks), higher net fluid balance at discharge (−138 vs. −786 mL), and higher prevalence of chronic hypertension (*n* = 17 vs. 7) compared to those in the nifedipine groups (*n* = 162). After controlling for confounders, incidence of readmission was 88% lower in nifedipine group (1.2%  vs. 8.1%); aOR of 0.12 (95% confidence interval [CI] 0.02 to 0.56). The frequency of severe HTN, acute treatment of HTN during hospitalization, use of magnesium sulfate and HTN in the 12 h prior to discharge was similar between groups.

**Conclusion and relevance:**

Primary outcome was statistically significant and consistent with observational data. Our findings support nifedipine as the first‐line agent for treatment of postpartum hypertension.

## 1 BACKGROUND

In the United States, 60% of pregnancy‐related deaths due to hypertension (HTN) occur in the postpartum period [1, 2, 3]. The impact of HTN is likely underestimated, as it is both an independent risk factor for maternal mortality and a mediator for other causes of morbidity and mortality such as cerebrovascular accident, myocardial infarction, vascular dissection, and cardiomyopathy [4, 5, 6, 7, 8, 9, 10, 11, 12, 13, 14, 15, 16, 17, 18, 19, 20]. Maternal Morbidity and Mortality Committees are now using chain of risk analysis and increasingly identifying HTN as an early risk factor in these causes of mortality that were previously analyzed as independent factors [21].

While there has been increased interest in postpartum HTN surveillance and its relationship to maternal morbidity, there is a paucity of studies evaluating optimal medical treatment or the impact of current treatments on maternal outcomes [22, 23, 24, 25, 26, 27, 28, 29]. As such, national and international guidelines for postpartum treatment of HTN can only provide expert opinion or recommend utilization of adult HTN guidelines due to the lack of evidence to guide blood pressure goals or medical management [30, 31, 32, 33].

To fill this void, multiple observational studies have been published and demonstrated nifedipine is associated with adequate blood pressure (BP) control and lower risk of readmission due to hypertensive complications [34, 35, 36, 37]. Randomized controlled trials completed to date evaluating various medications for HTN in the postpartum period have been underpowered, utilized composite outcomes, or had primary outcomes focused on short‐term results within the index hospitalization [38, 39, 40, 41, 42, 43, 44].

The purpose of this study was to determine if labetalol or nifedipine is superior in the management of postpartum HTN. We hypothesized nifedipine is superior to labetalol in the management of postpartum HTN based on observational studies and is associated with a reduced risk of postpartum hospital readmission. We designed a pragmatic open‐label, randomized controlled trial to test this hypothesis.

## 2 METHODS

This was a single institution, open‐label, randomized controlled trial at a suburban Level III Obstetric Hospital with a Level III Neonatal Intensive Care Unit. English‐speaking patients 19 years or older with two BPs > 140  mmHg systolic and/or > 90  mmHg diastolic at least 4 h apart during their admission for delivery were eligible for participation. Patients were identified by provider referral or by using an electronic medical record (EMR) screening tool that produced a daily report of patients meeting inclusion criteria. BP data were confirmed and exclusion criteria were evaluated by study personnel. Patients were excluded if they were already taking 2 BP medications, had contraindications to study medications, met inclusion the day of discharge, had baseline bradycardia < 60 or baseline tachycardia > 110. Written informed consent was obtained before allocation. Both block size and patient assignment (labetalol/nifedipine) were randomized using a publicly available research randomization tool. (Sealed Envelope Ltd. 2024, Create a blocked randomization list. [online] Available from htttps://sealedenvelope.com/simple‐randomiser/v1/lists [Accessed May 24, 2022].) Permuted block randomization was utilized. Sequence, block size, and patient number were unknown to research nurses performing patient enrollment. After enrollment, a central number was called to determine patient allocation. The type of HTN used in the analysis of clinical features was based on documented ICD10 codes (chronic HTN, preeclampsia with or without severe features, gestational HTN, and superimposed preeclampsia). The remainder of variables were obtained from review of patient data. The trial followed the Consolidated Standards of Reporting Trials (CONSORT) guidelines. A Data Safety Monitoring Board (DSMB) was established prior to enrolling patients and provided predetermined thresholds for discontinuation. Funding for research nurses was provided by the hospital's foundation which had no role in study design, data collection, data analysis, data interpretation, or writing of the manuscript.

### 2.1 Interventions and assessments

Participants were assigned to receive 30 mg nifedipine extended‐release twice daily (BID) or labetalol 200 mg every 8 h. We recognize the dosing of nifedipine is novel. In our clinical experience, patients on daily dosing had increased frequency of HTN in the 2–4 h before they were due for daily medication compared to those on the same total dose of nifedipine divided BID. The remainder of postpartum care was deferred to the admitting provider, including but not limited to escalation of therapy, addition of new medications, changing medications, medication frequency, monitoring blood work, timing of discharge, timing of follow‐up, and management of magnesium sulfate. Electronic medical records were reviewed 6‐ and 12‐weeks following delivery for clinical follow‐up and readmission data. Independent providers whose clinics are outside the hospital system EMR were contacted for data regarding primary outcome, postpartum follow‐up, medication management, and complications.

### 2.2 Primary outcome

The primary outcome for comparison was postpartum readmission less than 6 weeks after delivery for hypertensive complications. This outcome was selected due to the frequency of occurrence as well as noted parallels between readmission and mortality in the general medical literature [45, 46, 47]. Patients presenting with severe HTN, evidence of preeclampsia with severe features (Box 1), cardiomyopathy with HTN on presentation, stroke associated with HTN, myocardial infarction, and eclampsia, were considered hypertensive readmissions and included in the primary outcome. Patients presenting to clinic or the emergency department without evidence of severe features or with other non‐HTN related postpartum complications were not included in the readmission cohort. Since our primary outcome was used as a surrogate for maternal mortality, an independent DSMB reviewed the results of every 100 patients enrolled with the predetermined plan to terminate the trial if statistical significance reached *p* ≤ 0.01.

**Box 1: ACOG criteria for severe preeclampsia**

**Table:** 

**Blood Pressure**	Systolic ≥160 mmHg or diastolic ≥110 mmHg (on two occasions at least 4 h apart)
**Thrombocytopenia**	Platelet count < 100 × 10^9^/L
**Renal Insufficiency**	Serum creatinine > 1.1 mg/dL or doubling of creatinine
**Impaired Liver Function**	Elevated liver enzymes (AST/ALT > 2x normal)
	Severe persistent right upper quadrant or epigastric pain
**Pulmonary Edema**	Acute pulmonary edema or significant fluid retention
**Cerebral or Visual Symptoms**	New onset of cerebral or visual disturbances without alternative diagnosis (e.g., headaches, blurred vision)

### 2.3 Sample size calculation

The sample size was calculated using estimated readmission rates of 1% for nifedipine and 7% for labetalol based on a prior retrospective study of our patient population [36]. A power of 80% and alpha of 0.05 indicated 166 patients would need to be enrolled in each arm. Dilution of results was anticipated due to the low‐risk cohort, lower incidence of readmission in comparison to observational data, changes in the management of postpartum HTN at our institution, high rates of crossover, loss to follow up, and potential increased use of two medications. As a result, we estimated 300 patients would be required in each treatment arm for a total of 600 patients.

### 2.4 Statistical analysis

Analysis was stratified by assigned medication at randomization (intention‐to‐treat). Cohort characteristics were compared between patients randomized to labetalol versus nifedipine using univariate analysis. Categorical variables were compared using the chi‐square test. Normal distribution was determined in linear variables using Shapiro–Wilk test. Normally distributed linear variables were compared using the *t*‐test, otherwise the rank‐sum test was used.

The primary outcome of interest was compared between labetalol and nifedipine using chi‐square test. Multivariate logistic regression was performed to further evaluate the comparative effectiveness of the antihypertensive medications adjusting for clinically relevant variables that failed randomization. The regression model included adjustment for pre‐pregnancy BMI, gestational age at delivery, chronic HTN, number of elevated BPs during admission, and fluid balance at discharge. Statistical analysis was performed using Stata 18 (College Station, TX, USA)

## 3 RESULTS

### 3.1 Study population

**FIGURE 1:**
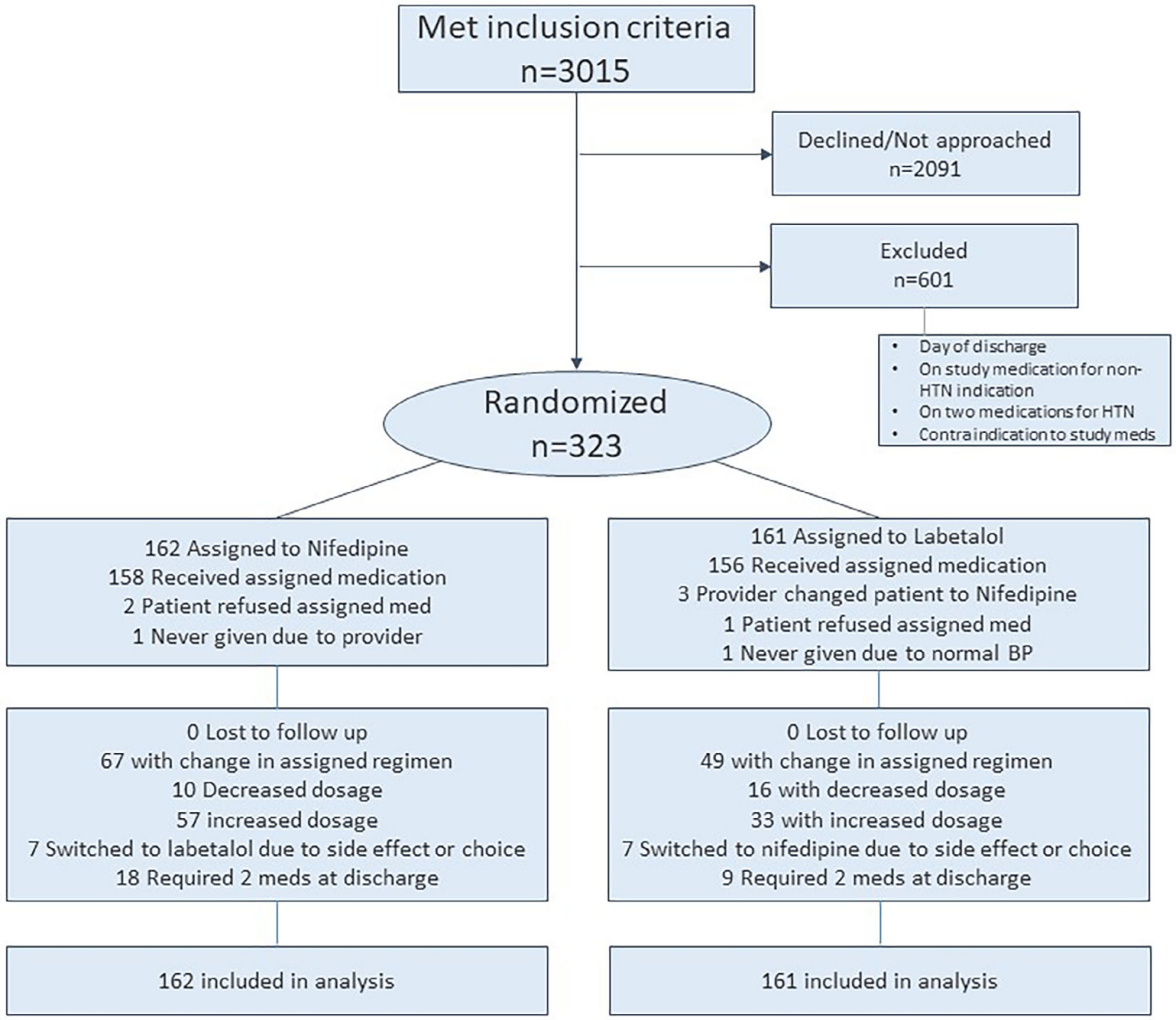
Consort flow diagram.

From July 2022 to July 2024, 3015 patients met the inclusion criteria. A total of 323 patients were enrolled in the study of which 161 were randomized to labetalol 200  mg every 8 h and 162 were randomized to nifedipine extended release 30  mg twice daily (Figure 1). Patients randomized to labetalol had higher rates of chronic HTN (17/161 vs. 7/162, *p* value = 0.03), pre‐pregnancy BMI (34.9 vs. 32.4, *p* value = 0.01), and later delivery (38 weeks GA vs. 37 weeks GA, *p* value = 0.02) (Table 1). Those randomized to nifedipine had more elevated BP measurements (12 vs. 9, *p* value = 0.02) and greater diuresis at discharge (−786 vs. −138, *p* value 0.03) (Table 2).

**TABLE 1:** Cohort characteristics.

	Labetalol	Nifedipine	*p* value
*N*	161	162	
Maternal age, mean (SD), years	31.4 ± 4.9	31.1 ± 5.5	0.56
Parity, mean (SD)	2 [1–2]	1 [1–2]	0.063
Gestational age at delivery, mean (SD), weeks	38 [37–39]	37 [36–38]	0.019
ppBMI, mean (SD), kg/m^2^	31 [26–35.5]	29 [23.5–33.5]	0.01
BMI at delivery, mean (SD), kg/m^2^	34.9 [30.7–39.0]	32.4 [29.1–37.6]	0.01
Race/ethnicity, *n* (%)			
Caucasian	145 (90.1)	149 (92.0)	0.41
Non‐Hispanic Black	4 (2.5)	2 (1.2)	
Hispanic	6 (3.7)	8 (4.9)	
Asian	3 (1.9)	3 (1.9)	
Other	3 (1.9)	0	
Private Insurance, *n* (%)	138 (85.7)	140 (86.4)	0.98
Vaginal Delivery, *n* (%)	96 (59.6)	99 (61.1)	0.79
Multiple Gestation, *n* (%)	7 (4.4)	13 (8.0)	0.17
CHTN, *n* (%)	17 (10.6}	7 (4.3)	0.03
Diabetes Mellitus, *n* (%)			
GDMA1	9 (5.6)	16 (9.9)	0.12
GDMA2	13 (8.1)	6 (3.7)	
Type I	1 (0.6)	4 (2.5)	
Type II	0	1 (0.3)	
HDP prior to admission, *n* (%)	100 (62.1)	108 (66.7)	0.39
Primary Provider, *n* (%)			
CNM	12 (7.5)	10 (6.2)	0.69
MFM	12 (7.5)	16 (9.9)	
OB	137 (85.1)	136 (84.0)	
eQBL at delivery, mean [SD], mL	325 [140–620]	400 [200–615]	0.1
ASA antepartum, *n* (%)	55 (34.6)	46 (28.6)	0.25
Tobacco use/Vaping, *n* (%)	7 (4.4)	7 (4.4)	0.98

Abbreviations: BMI, body mass index; CHTN, chronic hypertension; CNM, certified nurse midwife; GDMA, gestational diabetes mellitus white classification A1 and A2; HDP, hypertensive disease of pregnancy; MFM, maternal fetal medicine; OB; obstetrician; ppBMI, pre‐pregnancy body mass index.

**TABLE 2:** Clinical characteristics.

	Labetalol *n* = 161	Nifedipine *n* = 162	*p* value
Number of elevated BPs, mean [SD]	9 [5–17]	12 [7–20]	0.02
Any severe HTN, *n* (%)	76 (47.2)	87 (53.7)	0.24
Two or more severe BP, *n* (%)	51 (31.7)	56 (34.6)	0.58
Acute treatment of HTN, *n* (%)	38 (23.6)	39 (24.1)	0.92
# of acute treatments, mean [SD]	1.6 [0‐4]	1.6 [0–5]	0.9
Superimposed pre‐eclampsia, *n* (%)	3 (1.9)	2 (1.2)	0.65
HTN in the 12 h prior to discharge, *n* (%)	62 (38.5)	57 (35.2)	0.54
Magnesium sulfate, *n* (%)	38(23.6)	39(24.1)	0.46
Fluid balance at discharge, mean [SD], mL	−138 [−2220 to 1390]	−786 [−3312 to 735]	0.03

Of those with CHTN, five entered the study on antihypertensive medication. Three patients randomized to the nifedipine arm and two to the labetalol arm. Two patients changed medications, both from labetalol to nifedipine. Only one patient with CHTN on medication was readmitted. This patient was taking labetalol and randomized to the labetalol arm.

Those randomized to the labetalol arm that were readmitted had evidence of significant cardiac strain (mean pro‐BNP 905), abnormal lab studies, and severe HTN despite reported medication adherence. In contrast, the two readmissions in the nifedipine arm were associated with acute cholecystitis and reported medication non‐compliance the day of discharge (Table 3). Three patients in the labetalol arm and one in the nifedipine arm were not admitted at the time of their evaluation in the emergency department but based on their presentation, objective patient data, and our hospital's guidelines, should have been readmitted for observation due to the presence of severe hypertensive disease and, thus, were included in the readmission cohort.

**TABLE 3:** Readmitted patient characteristics.

Study medication	Prescribed medication	D/C to readmit (days)	Indication for admit	Readmit LoS (days)	Platelet (10^3^/uL)	Creatinine (mg/dL)	AST (U/L)	ALT (U/L)	Pro‐BNP (pg/mL)	CXR	Mat Echo
Labetalol	None	2	SOB, proBNP	2	209	0.9	**106**	**118**	**962**	–	WNL
Labetalol	Labetalol	9	HA, sHTN	2	289	0.64	23	24	**–**	–	
Labetalol	Labetalol	9	sHTN	4	294	0.86	22	17	**–**	–	
Labetalol	Labetalol	4	sHTN, HA	2	394	0.45	36	**60**	**–**	–	
Labetalol	Labetalol	4	sHTN	1	314	**1.17**	17	12	**552**	Pulm edema	WNL
Labetalol	Labetalol	6	SOB, sHTN, CP	3	278	0.72	**69**	**73**	**379**	–	
Labetalol	Labetalol & Nifedipine	3	SOB, sHTN	5	539	**1.01**	22	**135**	**3170**	–	WNL
Labetalol	Labetalol	5	sHTN, HA	2	187	0.65	**70**	**69**	**339**	Pulm edema	
Labetalol	Labetalol	3	sHTN, HA	3	368	0.7	23	30	**–**	–	
Labetalol	Labetalol	5	sHTN	NA	256	0.66	27	37	**632**	Normal	
Labetalol	Labetalol	2	sHTN	3	320	**0.96**	16	14	**–**	–	
Labetalol	Labetalol	8	sHTN	NA	288	0.66	18	23	**244**	–	
Labetalol	Labetalol	6	sHTN, HA	NA	222	0.56	41	32	–	–	
Nifedipine	Nifedipine	2	RUQ Pain, sHTN	NA	333	0.58	15	22	10	–	
Nifedipine	Nifedipine	0	sHTN	1	301	0.57	15	17	–	–	

Abbreviations: ALT, alanine aminotransferase; AST, Aspartate aminotransferase; CP, chest pain; D/C, discharge; Echo, echocardiogram; ED, emergency department; HA, Headache; LoS, length of stay; mHTN, mild hypertension; pro‐BNP, N‐Terminal Pro Brain Natriuretic Peptide; RUQ, right upper quadrant; sHTN, severe hypertension; SOB, shortness of breath; WNL, within normal limits.

### 3.2 Primary outcome

After the enrollment of 300 patients, the DSMB reviewed the data and determined we had reached the predetermined threshold of significance and the study was terminated. At the time the study was discontinued, 323 patients had enrolled and all those enrolled were included in the intent‐to‐treat analysis.

There was a total of 24 patients with postpartum readmission events identified at office visits or on presentation for emergency department evaluation due to postpartum complications. Of those, 63% (15/24) were deemed to be related to hypertensive complications. There were three adverse events within the study. Two patients in the labetalol arm reported dizziness and one patient in the nifedipine arm reported facial swelling and headache.

In the labetalol group, 13 out of 161 (8.1%) experienced a primary outcome event, compared to 2 out of 162 (1.2%) in the nifedipine group (*p* value = 0.003). After adjusting for clinically relevant population characteristics, the adjusted odds ratio for nifedipine versus labetalol was 0.12, 95% CI (0.02–0.56). Based on these results, 15 patients would need to be placed on nifedipine instead of labetalol to prevent 1 readmission event (NNT 14.6 [8.8–43.5]) (Table 4).

**TABLE 4:** Primary outcome.

	Labetalol *n* = 161	Nifedipine *n* = 162	*p* value	Adjusted odds ratio [95% CI]^a^
Readmission for Hypertensive Complication, *n* (%)	**13 (8.1)**	**2 (1.2)**	**0.003**	**0.12 [0.02–0.56]**
Number Needed to Treat, *n* [95% CI]	**14.6 [8.8‐43.5]**			

^a^ Logistic regression: adjusted for pre‐pregnancy BMI, gestational age, chronic hypertension, number of elevated BPs and fluid balance at discharge.

### 3.3 Comment

This single‐center randomized controlled trial demonstrated that in women with postpartum HTN, nifedipine administered twice daily reduced the risk of hospital readmission due to severe hypertensive complications by 88% when compared to labetalol three times daily. Consistent with other studies indicating improved diuresis with the use of nifedipine, discharge diuresis was 650 mL greater, providing at least one mechanism for how nifedipine may improve postpartum outcomes [48].

Further study is needed to confirm these findings and identify second‐line agents for patients with contraindications or inability to tolerate nifedipine, such as angiotensin converting enzyme inhibitors and diuretics [38, 39, 40, 41, 42, 43, 44, 49]. In addition to identifying equivalent or safer medications, steps need to be taken to determine optimal blood pressure goals in the postpartum period. Several attempts have been made to evaluate the optimal BP goal in the postpartum period with mixed results. Given our finding nifedipine significantly reduces readmission risk, those studies are now limited in their conclusions as they did not control for the type of antihypertensive used for postpartum BP management, potentially impacting their analyses [36, 50, 51, 52]. Last, studies should be performed evaluating how medications with varying mechanisms of action perform as a second agent in patients with refractory postpartum HTN requiring more than nifedipine. With the inferiority of labetalol demonstrated in this study and the frequent use of labetalol in postpartum HTN, evaluating its safety as a second agent should also be an early objective of future studies.

### 3.4 Strengths and limitations

The strengths of the study include the use of patient‐level data, pragmatic study design allowing for patient care within patterns of normal care, easily reproduced inclusion criteria, use of clinically established criteria, inclusion of relevant confounding variables, outcome measures of clinical significance, and a study design allowing ready adaptation to already established HTN safety bundles [53].

Limitations of the study include its performance at a single center, uniform population, lack of blinding, heterogeneity of management, overestimation of needed enrollment, unrecognized confounding variables, and the early closure after reaching a predetermined statistical significance. Since we left ongoing management in the hands of the primary provider, we anticipated that a significant number of patients would be changed to the provider's preferred medication, impacting the power of the study. Only seven participants changed medications due to side effects or primary provider's preference when we expected this to be closer to 10% or 15% of all patients. Despite this overestimation, it is worth noting that the number of patients enrolled at the time of termination was consistent with the original power calculation.

## 4 CONCLUSION

The results of this trial corroborate observational data indicating nifedipine is a significantly safer antihypertensive medication in postpartum HTN than labetalol and supports the use of nifedipine as the first‐line agent for management of postpartum HTN.

## CONFLICT OF INTEREST STATEMENT

The authors report no conflicts of interest.
